# Collagen type I-mediated mechanotransduction controls epithelial cell fate conversion during intestinal inflammation

**DOI:** 10.1186/s41232-022-00237-3

**Published:** 2022-11-28

**Authors:** Sakurako Kobayashi, Nobuhiko Ogasawara, Satoshi Watanabe, Yosuke Yoneyama, Sakura Kirino, Yui Hiraguri, Masami Inoue, Sayaka Nagata, Yoshimi Okamoto-Uchida, Satoshi Kofuji, Hiromichi Shimizu, Go Ito, Tomohiro Mizutani, Shinichi Yamauchi, Yusuke Kinugasa, Yoshihito Kano, Yasuhiro Nemoto, Mamoru Watanabe, Kiichiro Tsuchiya, Hiroshi Nishina, Ryuichi Okamoto, Shiro Yui

**Affiliations:** 1 Department of Gastroenterology and Hepatology, Tokyo Medical and Dental University (TMDU), 1-5-45 Yushima, Bunkyo-ku, Tokyo, 113-8510 Japan; 2 Institute of Research, Tokyo Medical and Dental University (TMDU), 1-5-45 Yushima, Bunkyo-ku, Tokyo, 113-8510 Japan; 3 Department of Developmental and Regenerative Biology, Medical Research Institute, Tokyo Medical and Dental University (TMDU), 1-5-45 Yushima, Bunkyo-ku, Tokyo, 113-8510 Japan; 4 Center for Stem Cell and Regenerative Medicine, Tokyo Medical and Dental University (TMDU), 1-5-45 Yushima, Bunkyo-ku, Tokyo, 113-8510 Japan; 5 Advanced Research Institute, Tokyo Medical and Dental University (TMDU), 1-5-45 Yushima, Bunkyo-ku, Tokyo, 113-8510 Japan; 6 Department of Gastrointestinal Surgery, Tokyo Medical and Dental University (TMDU), 1-5-45 Yushima, Bunkyo-ku, Tokyo, 113-8510 Japan; 7 Department of Clinical Oncology, Graduate School of Medical and Dental Sciences, Tokyo Medical and Dental University (TMDU), 1-5-45 Yushima, Bunkyo-ku, Tokyo, 113-8510 Japan; 8 Department of Gastroenterology, Faculty of Medicine, University of Tsukuba, 1-1-1 Tennoudai, Tsukuba, Ibaraki, 305-8575 Japan

**Keywords:** YAP/TAZ-TEAD axis, Fetal-like reprogramming, Regenerative and inflammatory response, Inflammatory bowel disease, Mechanotransduction

## Abstract

**Background:**

The emerging concepts of fetal-like reprogramming following tissue injury have been well recognized as an important cue for resolving regenerative mechanisms of intestinal epithelium during inflammation. We previously revealed that the remodeling of mesenchyme with collagen fibril induces YAP/TAZ-dependent fate conversion of intestinal/colonic epithelial cells covering the wound bed towards fetal-like progenitors. To fully elucidate the mechanisms underlying the link between extracellular matrix (ECM) remodeling of mesenchyme and fetal-like reprogramming of epithelial cells, it is critical to understand how collagen type I influence the phenotype of epithelial cells. In this study, we utilize collagen sphere, which is the epithelial organoids cultured in purified collagen type I, to understand the mechanisms of the inflammatory associated reprogramming. Resolving the entire landscape of regulatory networks of the collagen sphere is useful to dissect the reprogrammed signature of the intestinal epithelium.

**Methods:**

We performed microarray, RNA-seq, and ATAC-seq analyses of the murine collagen sphere in comparison with Matrigel organoid and fetal enterosphere (FEnS). We subsequently cultured human colon epithelium in collagen type I and performed RNA-seq analysis. The enriched genes were validated by gene expression comparison between published gene sets and immunofluorescence in pathological specimens of ulcerative colitis (UC).

**Results:**

The murine collagen sphere was confirmed to have inflammatory and regenerative signatures from RNA-seq analysis. ATAC-seq analysis confirmed that the YAP/TAZ-TEAD axis plays a central role in the induction of the distinctive signature. Among them, TAZ has implied its relevant role in the process of reprogramming and the ATAC-based motif analysis demonstrated not only Tead proteins, but also Fra1 and Runx2, which are highly enriched in the collagen sphere. Additionally, the human collagen sphere also showed a highly significant enrichment of both inflammatory and fetal-like signatures. Immunofluorescence staining confirmed that the representative genes in the human collagen sphere were highly expressed in the inflammatory region of ulcerative colitis.

**Conclusions:**

Collagen type I showed a significant influence in the acquisition of the reprogrammed inflammatory signature in both mice and humans. Dissection of the cell fate conversion and its mechanisms shown in this study can enhance our understanding of how the epithelial signature of inflammation is influenced by the ECM niche.

**Supplementary Information:**

The online version contains supplementary material available at 10.1186/s41232-022-00237-3.

## Background

Recent findings according to a robust regenerative response to severe damage demonstrate that the intestinal epithelium is an appropriate model organ for tissue regeneration [1]. The intestinal epithelium is composed of a single layer of intestinal epithelial cells lining the intestinal tract and possesses a distinctive crypt-villus architecture [2]. Stem cells, exhibiting both self-renewal and multipotent differentiation potentials, reside in the crypt bottom and play essential roles in maintaining tissue homeostasis [3]. We previously reported that the LY6 member stem cell antigen-1 (Sca1/Ly6a)-expressing fetal-like cells emerged in the swelling areas of the repairing epithelium in the dextran sulfate sodium (DSS)-induced colitis mouse model [4]. The transient fetal-like reprogramming was subsequently reported in other studies: the occurrence of Sca1-positive cells following the disruption of the epithelial integrity by parasite infection [5], or the expansion of clusterin (Clu)-positive cells following the intestinal damage by irradiation and DSS colitis [6]. These cells are called as regenerative stem cells being apart from Lgr5 stem cells and recognized as an essential cell population which is in charge of tissue regeneration [7], and the emerging concepts of fetal-like reprogramming have been well recognized as important cues for resolving regenerative mechanisms of the intestinal epithelium [8, 9]. In order to facilitate our knowledge how inflammation enhances regeneration, it is important to dissect the mechanisms of the reprogramming towards a primitive state.

We previously reported that collagen deposition in mesenchyme during inflammation activates YAP/TAZ in the epithelial compartment overlying the wound bed, forming a regenerative cascade to induce fetal-like epithelial cells during intestinal inflammation. Accordingly, the in vitro culture system of murine intestinal epithelium embedded in collagen type I under serum-free medium [10] can recapitulate the fetal-like reprograming seen in intestinal inflammation, and the cystic organoids established under the culture system exhibit the acquisition of reprogrammed and regenerative phenotype [4]. This scheme is one of the key findings to dissect how inflammation triggers the regenerative response; however, the details of transcriptomic and epigenetic mechanisms governing the fetal-like reprogramming in intestinal epithelium remain to be elucidated. It is also important to adapt the culture system to human samples to identify the ECM-dependent epithelial cell plasticity in the human case.

In the current study, we performed a detailed analysis of the murine collagen sphere to determine the transcriptional dynamics and epigenetic characteristics to understand collagen type I-mediated mechanotransduction. Subsequently, we applied the collagen culture system to the human colon and revealed that the fate conversion of epithelial cells towards both an inflammatory and a primitive state can be recapitulated in human epithelial cells. Furthermore, we validated the expression of representative genes identified through the character analysis of the collagen sphere in vitro and in vivo. Overall, the collagen sphere was revealed to reproduce a part of the inflammatory response in both mice and humans. These results suggest that collagen type I functions as an important niche for tissue regeneration by converging the epithelial phenotype towards a fetal-like regenerative population. The collagen sphere is a useful methodology not only to dissect a distinct epithelial cell population induced during tissue inflammation but also to provide a better resolution in understanding pathology/pathophysiology commonly underlying in various types of human intestinal inflammation such as inflammatory bowel disease (IBD).

## Materials and methods

### Mice

C57BL/6J mice were purchased from CLEA Japan (Tokyo, Japan). Animals from embryonic day 16 (E16) to adulthood (12 weeks) were used for in vitro cultures. Adulthood animals are female, and the gender of the embryo is not identified.

### Primary cultures of adult murine small intestinal epithelium in Matrigel or collagen type I

The establishment of organoids was performed as previously described [11]. Harvested crypts were suspended in 30μl Matrigel (Corning, #356231) or cellmatrix type IA (Collagen Type I, Nitta Gelatin, #631-00651) and plated onto a 24-well plate (Flat bottom; Corning, #3524). Advanced DMEM/F12 (Gibco, #12634-010) with GlutaMAX (Gibco; 1% v/v, #35050-061) and penicillin/streptomycin (Nacalai Tesque; 1% v/v, #26253-84) was used as basal medium. Culture medium contains murine EGF (PeproTech, #315-09), murine Noggin (R&D Systems, #1967-NG), mouse R-spondin1 (R&D Systems, #3474-RS), and N-2 (Gibco, #17502-048)/B-27 supplement (Gibco, #17504-044) (ENR) (Table 1) [12]. When indicated, murine Wnt3a (R&D Systems, #1324-WN), nicotinamide (Sigma-Aldrich, #72340-250G), and bovine serum albumin (BSA; Sigma-Aldrich, # A9576) were supplemented to ENR medium (ENRWN) (Table 1). The medium was subsequently changed every 2–3 days. Y-27632 (TOCRIS; 10μM, #1254) was supplemented for the first 2–3 days after passage.

**Table 1:** Culture conditions: culture conditions for mouse and human organoids are summarized in table

	Species	Mouse				Human	
	Segments	Distal small intestine				Colon	
	Ages	Adult			Fetal	Adult	
	Medium	ENR	ENRWN	ENRWN	ENRWN	WRN CM	Recombinant
	ECM	Matrigel	Matrigel	COL	Matrigel	Matrigel	COL
**Murine EGF**	50ng/ml	●	●	●	●	●	●
**Mouse Noggin**	100ng/ml	●	●	●	●		●
**Mouse R-spo1**	500ng/ml	●	●	●	●		●
**Mouse Wnt3a**	100ng/ml		●	●			
	300mg/ml						●
**WRN-CM**	20% v/v					●	
**Nicotinamide**	10mM		●	●		●	●
**N2**	1% v/v	●	●	●	●	●	●
**B27**	2% v/v	●	●	●	●	●	●
**BSA**	1% v/v		●	●			●
**Prostaglandin E2**	2.5μM						●
**NAC**	1mM					●	
**Gastrin I**	10nM					●	
**A83-01**	500nM					●	

*Abbreviations*: *BSA* bovine serum albumin solution in 30% DPBS, *CM* conditioned medium, *COL* collagen type I, *ECM* extracellular matrix, *R-spo1* R-spondin1

### Primary culture of human colonic epithelial cells in Matrigel or collagen type I

Human colonic crypts were harvested from surgical specimens. The epithelium was dissected and cut into small pieces. The fragments were washed in a 30-ml phosphate buffer saline (PBS; Nacalai Tesque, #14249-24) twice and subsequently in 2% Mucofilin (Eisai) once, followed by 4°C incubations in 15-mM EDTA for 20 min. After being washed in 30ml PBS, the tissues were incubated in 10-ml collagenase solution (Sigma-Aldrich; 6.25mg/ml in PBS, #C7657-500MG) at 37°C for 20 min. After settling down, the supernatant was transferred into another 50-ml conical tube by passing through a 70-μm pore size mesh filter. The total volume was adjusted to 10 mL with 0.1% v/v BSA/PBS into a 15-ml conical tube, and cells were pelleted at 500 g for 3 min. The pellets were suspended in Matrigel and plated onto a 24-well plate (Flat bottom; Corning, #3524). The basal medium is the same with murine culture, and supplementations are murine EGF, L-WRN-conditioned medium, nicotinamide, N-2/B-27supplement, *N*-acetyl-l cysteine (Sigma-Aldrich, #A9165-5G), Gastrin I (Sigma-Aldrich, #G9145), and A83-01 (Tocris, #2939) (Table 1). We passaged Matrigel organoids into collagen type I. The medium for collagen culture use the same basal medium with supplementation of murine EGF, murine Noggin, mouse R-spondin1, murine Wnt3a, nicotinamide, N-2/B-27 supplement, prostaglandin E2 (Nacalai Tesque, #29334-21), and BSA (Table 1).

### Primary culture of fetal intestinal epithelial cells in Matrigel

The establishment of fetal intestinal organoids from the distal part of the small intestine (dFEnS) was performed as previously described [13]. The isolated epithelium was embedded in Matrigel and cultured in ENRWN (Table 1).

### Flow cytometry

Adult murine intestinal organoids in Matrigel/collagen type I and dFEnS were dissociated into single cells by incubating fragmented organoids in 50% v/v TryPLE Express (Gibco, #12605-101) in advanced DMEM/F12 for 10–15 min. These single cells were stained with FITC anti-mouse CD326 (Ep-CAM) (Clone G8.8, BioLegend, 1:500, #118207), PE anti-mouse Ly6A/E (Sca-1) (Clone D7, BioLegend, 1:5000, #108107), and propidium iodide (Immunostep, 1:1000, #PI) before flow cytometry analysis with FACS Melody (BD Biosciences, Franklin Lakes, N). Live single cells were gated to assess for Sca-1 and Ep-CAM.

### Histology, imaging, and immunofluorescence

Organoids and surgical tissues were fixed with 4% paraformaldehyde (Nacalai Tesque, #09154-85) overnight. The samples were embedded in Tissue-Tek O.C.T. Compound (Sakura, #45833) and frozen, and 8μm-thick cryosections were prepared by Tissue-Tek Polar Microtome/Cryostat Polar (Sakura). Rat anti-YAP (Millipore, #MABS2029), rabbit anti-IL18 (Abcam, #ab243091), rabbit anti-KRT80 (Proteintech, #16835-1-AP), and recombinant anti-C4 binding protein/C4BPB antibody (Abcam, #ab199430) staining were performed. Nuclei were counterstained with VECTASHILED mounting medium (Vector, #H-1200). Images were captured with a Leica TCS SP8 confocal microscope and were analyzed in Fiji and Adobe Photoshop CS6.

### RNA extraction and cDNA synthesis

The total RNA of organoids was isolated using the RNeasy micro kit (Qiagen, #74004) according to the manufacturer’s protocol. cDNA was synthetized from 1000ng total RNA using superscript III reverse transcriptase (Invitrogen, # 18080044) and random primers.

### qRT-PCR analysis and visualization

Each reverse-transcription product was subjected to a PCR reaction using SYBR green master mix (QIAGEN, #20415), which was run on StepOnePlus Real-Time PCR system (Applied Biosystems). Each assay was performed in triplicate using specific primer sequences. The expression level was determined by ∆CT method, and mRNA levels were normalized to that of *Cdh1*. Fold changes relative to control (MG-ENR) were determined within each replicate and were visualized using heatmapper (http://www.heatmapper.ca) [14].

### DNA microarray analysis

The DNA microarray analysis of murine organoids was performed with a 3D-Gene Mouse Oligo chip 24k (Toray Industries Inc., Tokyo, Japan). This microarray adopted a columnar structure to stabilize spot morphology and to enable micro beads agitation for efficient hybridization. The total RNA was labeled with Cy5 by using the Amino Allyl MessageAMP II aRNA Amplification Kit (Applied Biosystems, CA, U.S.A.). The Cy5-labeled anti-sense RNA pools were mixed with a hybridization buffer and hybridized for 16 h according to the supplier’s protocols (www.3d-gene.com). The hybridization signals were acquired by using a 3D-Gene Scanner (Toray Industries Inc., Tokyo, Japan) and processed by 3D-Gene Extraction software (Toray Industries Inc., Tokyo, Japan). The signal intensity for each gene was normalized by the global normalization method (the median of the detected signal intensity was adjusted to 25). Pairwise comparisons of the normalized signal intensity were performed between each sample. Upregulated genes were identified based on fold change ≥2.

### RNA sequencing analysis

RNA sequencing analysis of murine organoids was performed by Active Motif. The total RNA was isolated from the cells using the Qiagen RNeasy Mini Kit (Qiagen). For each sample, 2μg of the total RNA was used in Illumina’s TruSeq Stranded mRNA Library kit. Libraries were sequenced on Illumina NextSeq 500 as paired-end 42-nt reads. Sequence reads were estimated as FPKM (fragments per kilobase of transcript per million mapped reads). A differentially expressed genes (DEGs) analysis was performed using the STAR alignment-DESeq2 software pipeline following.

RNA sequencing analysis of human organoids was performed by TaKaRa Bio, Inc. (Kusatsu, Japan). The total RNA was reversed transcribed into cDNA with a Clontech SMART-Seq v4 Low Input RNA Kit according to the manufacturer’s instructions. RNA sequencing libraries were constructed from the amplified cDNA using Illumina Nextera XT DNA Library Prep Kits, validated using an Agilent 4200 TapeStation, and sequenced on the Illumina NovaSeq 6000 platform. Gene expression level was estimated as TPM (transcripts per million) for principal component analysis (PCA). Subsequently, the raw read counts were normalized by relative log normalization (RLE) and differentially expressed analysis was conducted with DESeq2 (Version 1.24.0).

### Principal component analysis plot

PCA plots were generated using Partek genomic suite (version 7.19.1125) with normalized signal intensity in microarray of murine organoids, FPKM values in RNA sequencing of murine organoids, and TPM values in RNA sequencing of human organoids.

### Gene Ontology and Kyoto Encyclopedia of Genes and Genomes Analysis

Gene Ontology (GO) and Kyoto Encyclopedia of Genes and Genomes (KEGG) pathway enrichment analysis were performed and visualized based on DEGs using the Bioconductor package, clusterProfiler4.0 [15] in R software.

### Gene network analysis

Gene network analysis was performed by Cytoscape software (Version 3.9.1) using the String app (https://cytoscape.org).

### ATAC sequencing analysis

Cryopreserved cells were sent to Active Motif to perform the ATAC-seq assay. The cells were then thawed in a 37°C water bath, pelleted, washed with cold PBS, and tagmented as previously described, with some modifications based on the previous protocol. Briefly, cell pellets were resuspended in lysis buffer, pelleted, and tagmented using the enzyme and buffer provided in the Nextera Library Prep Kit (Illumina). Tagmented DNA was then purified using the MinElute PCR purification kit (Qiagen), amplified with 10 cycles of PCR, and subsequently purified by Agencourt AMPure SPRI beads (Beckman Coulter). The resulting material was quantified by the KAPA Library Quantification Kit for Illumina platforms (KAPA Biosystems) and sequenced with PE42 sequencing on the NextSeq 500 sequencer (Illumina). Reads were aligned using the BWA algorithm with default settings. Only reads that passed Illumina’s quality filter, aligned with no more than two mismatches, and mapped uniquely to the mouse genome (mapping quality≥1) were used in the subsequent analysis. Duplicate reads were removed. Alignments were extended in silico at their 3’-ends to a length of 200 bp and assigned to 32-nt bins along the genome to smooth the data. The resulting histograms (genomic “signal maps”) were stored in bigWig files. Peaks were identified using the MACS 2.1.0 algorithm at a cutoff of *p* value 1E^-7^, without control file, and with the –nomodel option. Peaks that were on the ENCODE blacklist of known false ChIP-Seq peaks were removed. Signal maps and peak locations were used as input data to Active Motifs proprietary analysis program, which creates Excel tables containing detailed information on sample comparison, peak metrics, peak locations, and gene annotations [16, 17].

### Statistical analyses

Statistical assessment of overlap between different gene sets was done using Fishers’ exact *T* test with the assumption that the number of total genes is 25,000 in both mice and humans.

## Results

### ECM directs the acquisition of developmental signature in vitro

**Fig. 1:**
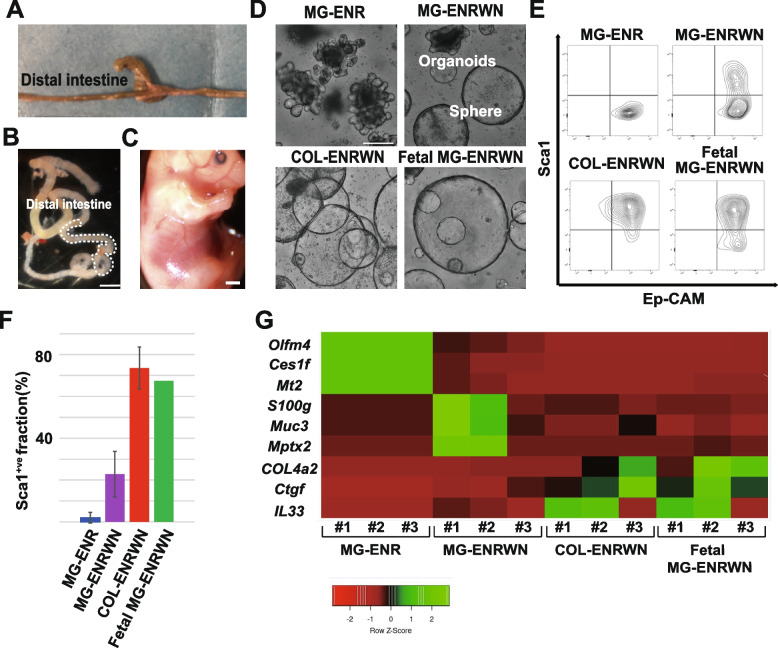
Extracellular matrix (ECM) directs the acquisition of developmental and regenerative signature in vitro. **A** The anatomy of the murine distal small intestine to establish intestinal organoids is shown. **B** A macroscopic image of the fetal murine intestine used to establish dFEnS is shown. The demarcated area indicates the distal small intestine used for in vitro expansion. Scale bar, 1mm. **C** A macroscopic image of the fetal mouse at E16 is shown. Scale bar, 1 mm. **D** Representative images of adult murine distal small intestinal organoids cultured in Matrigel with ENR medium (top left panel), in Matrigel with ENRWN medium (top right panel), in collagen with ENRWN medium (bottom left panel), and fetal murine intestinal organoids in Matrigel with ENRWN medium (dFEnS) (bottom right panel). Scale bar, 200μm. **E** Flow cytometric analysis of cells from the intestinal organoids in MG-ENR (top left panel), MG-ENRWN (top right panel), COL-ENRWN (bottom left panel), and fetal MG-ENRWN (dFEnS) (bottom right panel) is shown. Diagrams show representative plots for Sca1 in the live EpCAM+ve cell population. **F** The percentages of Sca1^+ve^ cells in MG-ENR, MG-ENRWN, COL-ENRWN, and fetal MG-ENRWN organoids are indicated. The diagram shows the average ± S.D. (*n*=3) for adult intestinal organoids grown in MG-ENR, MG-ENRWN, and COL-ENRWN, and the average (*n*=2) for fetal MG-ENRWN organoids (dFEnS). **G** qPCR analysis of different cultures (MG-ENR, MG-ENRWN, COL-ENRWN, and fetal MG-ENRWN (dFEnS)) of the small intestine in biological triplicates for indicated genes are shown as a heatmap

We firstly focused on the terminal ileum which is frequently affected by inflammation in the intestine [18]. We used a distal part of the small intestine; 3-cm length from the terminal ileum in adult mice and a distal 1/3 part between the cecum and pyloric ring in fetal mice at embryonic day 16 (E16) (Fig. 1A, B, C). For directly assessing the effect of ECM composition on epithelial cells, we took advantage of the organoid culture system enabling the expansion of intestinal epithelial cells embedded in the proper ECM with intestinal-specific pleiotropic factors [19]. As ECM, we compared Matrigel (MG) mainly consisting of laminin/collagen type IV and purified collagen type I. Harvested crypts were embedded in Matrigel under the ENR/ENRWN medium or in collagen type I gel (COL) under the ENRWN medium (Table 1). Organoids cultured in MG-ENR condition showed crypt-like budding structures [12]. The addition of Wnt3a and nicotinamide to standard Matrigel cultures, “MG-ENRWN” promoted a spherical phenotype, but with occasional budding domains as reported [4, 20]. In contrast, almost of all organoids cultured in COL-ENRWN condition showed spherical phenotype. The in vitro expansion of fetal crypts as spheroids (dFEnS) was promoted in MG-ENRWN condition (Fig. 1D).

Sca1 expression is positive in not only fetal intestinal epithelium cells but also fetal-like cells emerging in the injured epithelium during inflammation and following repairing phase [4, 5]. We performed Sca1 expression profiling on MG-ENR, MG-ENRWN, COL-ENRWN, and dFEnS using flow cytometric analysis (Fig. 1E). Percentages of Sca1 positive cells were 2%±2.5%, 22.8%±10.9%, and 73.6%±10.1% (average of biological triplicate with standard deviation) in MG-ENR, MG-ENRWN, and COL-ENRWN, respectively (Fig. 1F). As for dFEnS, the percentage of Sca1-positive cells was 67.5% (average of biological duplicate) (Fig. 1F).

We additionally performed a microarray analysis of the transcriptional profile of each organoid with the global normalization method (*N*=1). We analyzed the changes in 6597 gene expression after excluding the probe sets that failed to reach an expression value of 100 in at least 1 sample (Supplementary Table 1, Sheet 1). Principal component analysis (PCA) revealed that COL-ENRWN and dFEnS were plotted closely, while away from Matrigel organoids. Moreover, MG-ENRWN was away from both COL-ENRWN/dFEnS and MG-ENR (Supplementary Fig. 1A). After the selection of marker genes differentially expressed in each sample, we evaluate the expression level of *Olfm4, Ces1f*, and *Mt2* for MG-ENR, *S100g*, *Muc3*, and *Mptx2* for MG-ENRWN, and *Col4a2*, *Ctgf*, and *Il33* for COL-ENRWN/dFEnS by qPCR (*N*=3). The analysis clearly indicated that MG-ENRWN is a different group from COL-ENWRN/dFEnS (Fig. 1G).

Next, we compared the microarray data set with the Sca1-inflammatory signature, a gene set enriched in Sca1+ve cells sorted from DSS-treated animals with the threshold of fold change≥2, fdr<0.05 compared to homeostatic Ep-CAM positive population [4]. We revealed that only 8 of 265 upregulated genes in MG-ENRWN compared to MG-ENR (fold change≥2) (Supplementary Table 1, Sheet 2) overlapped with the Sca1-signature (*p*=3.2*10^-3^), whereas 25 of 380 upregulated genes in COL-ENRWN compared to MG-ENR (fold change≥2) (Supplementary Table 1, Sheet 3) overlapped with Sca1 signature (*p*=4.1*10^-14^) (Supplementary Fig. 1B, B’, C, C’), presenting that COL-ENRWN showed higher similarity with the Sca1 inflammatory signature than MG-ENRWN.

Together, these results indicate that the difference of ECM in culture (Matrigel or collagen type I) is the dominant factor in the acquisition of developmental and inflammatory phenotype over the medium composition, whether Wnt3a/nicotinamide is supplemented or not in the culture medium.

### Transcriptional characterization of collagen sphere

**Fig. 2:**
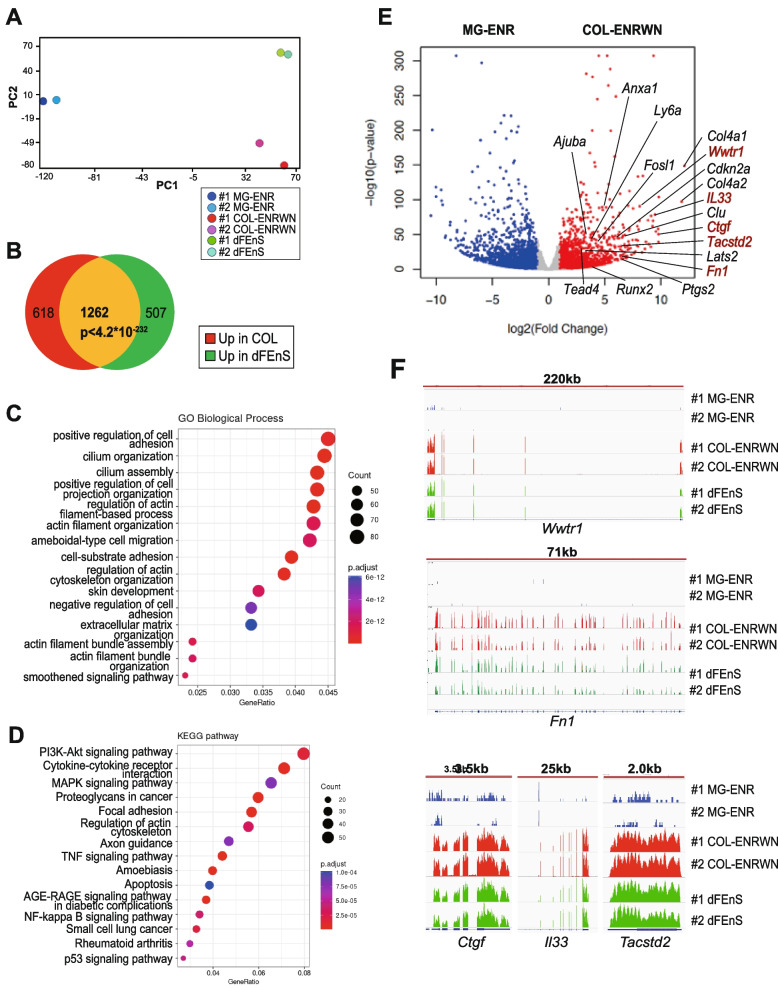
Transcriptional characterization of the collagen sphere. **A** Principal component analysis of RNA-seq data from MG-ENR, COL-ENRWN, and dFEnS based on the 12,137 variably expressed genes, of which adjust *p* value<0.1, is shown. **B** Venn diagram depicts the significant overlap of differentially expressed genes (DEGs) (padj<0. 1, FC≥2) between COL-ENRWN and dFEnS (*N*=2). Up in COL (red and yellow) indicates the numbers of genes upregulated in COL-ENRWN compared to MG-ENR (1880 genes); Up in dFEnS (green and yellow) represents the numbers of genes upregulated in dFEnS compared to MG-ENR (1769 genes). Total of 1262 genes are overlapped between these 2 gene sets (yellow). The *p* value of statistical significance is indicated. **C** Top 20 GO-TERM (BP direct) of upregulated gene set in COL-ENRWN compared to MG-ENR is shown. **D** Top 20 KEGG pathway enriched in upregulated gene set in COL-ENRWN compared to MG-ENR is shown. **E** A volcano plot of DEGs (padj<0. 1, FC≥2) between MG-ENR and COL-ENRWN with DEGs indicated. Each plot indicates the gene with the average log 2-fold change (*x*-axis) and -log10(*p* value) (*y*-axis) of duplicate samples of each condition. **F** The RNA sequencing tracks of *Wwtr1*, *Fn1*, *Ctgf*, *Il33*, and *Tacstd2* in MG-ENR (blue), COL-ENRWN (red), and dFEnS (green) are shown

After excluding MG-ENRWN, we applied RNA sequencing to analyze the transcriptional profile of the collagen sphere in comparison with MG-ENR and dFEnS (*N*=2). We identified a total of 12,137 DEGs using cutoff criteria of adjusted *p* value (padj)<0.1 (Supplementary Table 2, Sheet 1). Consistent with microarray analysis, COL-ENRWN was plotted close to dFEnS according to PCA (Fig. 2A).

We identified 1880 genes upregulated in COL-ENRWN compared to MG-ENR (COL gene set; Supplementary Table 2, Sheet 2), and 1769 genes upregulated in dFEnS compared to MG-ENR (fetal gene set; Supplementary Table 2, Sheet 3), using cutoff criteria of fold change≥2 and padj<0.1, respectively. COL gene set showed significant overlap with the fetal gene set (Fig. 2B), and a total of 1262 genes were upregulated in both COL-ENRWN and dFEnS compared to MG-ENR (*p*<4.2*10^-232^). In GO TERM (Biological process) analysis, positive regulation of cell adhesion, cilium organization, actin filament organization, skin development, extracellular matrix organization, etc., were related with the COL gene set (Fig. 2C). In KEGG enrichment analysis, PI3-Akt signaling pathway, proteoglycans in cancer, focal adhesion, TNF signaling pathway, amoebiasis, NF-κΒ signaling pathway, p53 signaling pathway, etc., were related with the COL gene set (Fig. 2D). COL gene set included genes such as *Col4a1/2* (fold change (FC): 4390/3641), *Ctgf* (FC: 750), *Il33* (FC: 709), *Wwtr1* (FC: 276), *Clu* (FC: 110), *Fn1* (FC: 84), *Cdkn2a* (FC: 74), *Ptgs2* (FC: 64), *Anxa1* (FC: 28), *Fosl1* (FC: 19), *Runx2* (FC: 18), *Ly6a* (FC: 13), *Ajuba* (FC: 9.4), *Lats2* (FC: 7.6), and *Tead4* (FC: 7.2) (Fig. 2E).

The posttranscriptional regulation, that is the nuclear accumulation of YAP/TAZ, is generally considered as a crucial step in the activation of YAP/TAZ [21]. We previously reported that the epithelial polarity was lost, and YAP nuclear localization was retained in the collagen sphere [4]. Accordingly, we confirmed that the expression level of *Yap1* between MG-ENR and COL-ENRWN is not different (shrunken Log FC=0.26, COL-ENRWN/MG-ENR) (Supplementary Table 2, Sheet 1). Interestingly, however, *Wwtr1*, which is a coding gene of TAZ playing redundant roles with YAP1 [22] as a Hippo mediator, is identified in the COL gene set as described above. This implies that transcriptional upregulation of *Wwtr1* is involved in the regenerative cascade mediating the cellular mechanoresponses in intestinal inflammation. Upregulation of *Tead4,* a cofactor of YAP/TAZ directly binding to DNA to form complex, also confirms that YAP/TAZ-TEAD axis plays a central role in mediating the collagen type I-dependent fate conversion. Additionally, activator protein1 (AP-1) transcriptional factor *Fosl1*, which forms a complex with YAP/TAZ/TEAD [23] and *Runx2* also known as a coactivator of YAP/TAZ [24, 25], was included in the COL gene set. The RNA sequencing tracks of *Wwtr1*, *Fn1*, *Ctgf*, *Il33*, and another fetal marker *Tacstd2* [26] clearly illustrated the change of transcriptional state (Fig. 2F).

The whole transcriptome analysis revealed that the collagen sphere is a useful inflammatory model representing the reprogrammed signature of epithelial cells and we think that the collagen sphere can be referred to as “inflammanoid.”

### Open chromatin status and transcriptional factors in the acquisition of inflammatory signature

**Fig. 3:**
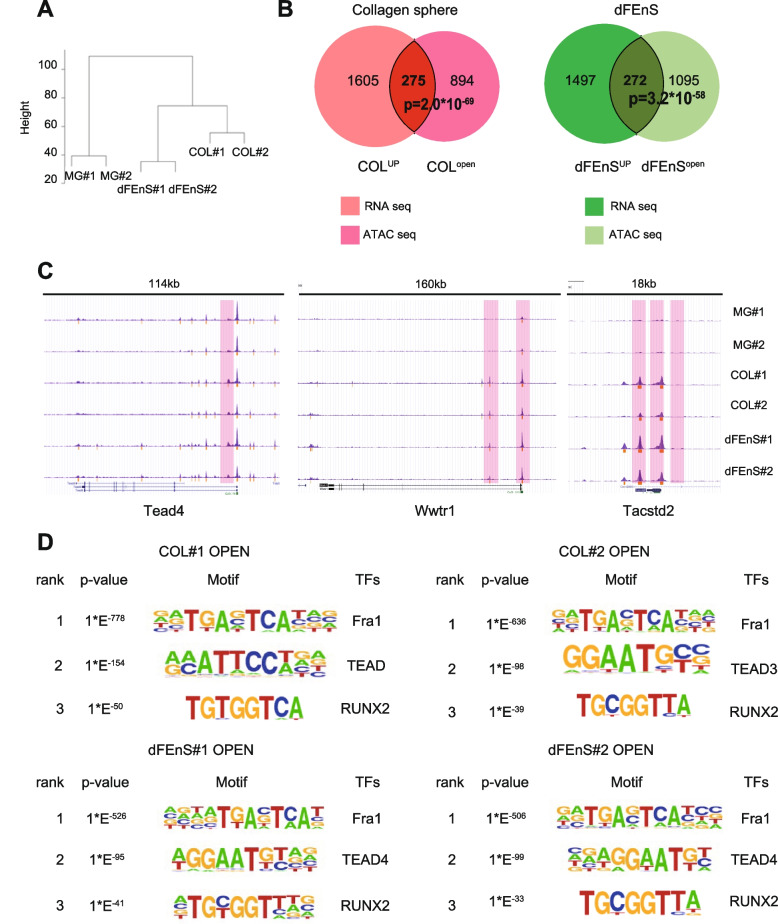
Open chromatin status and transcriptional factors in the acquisition of inflammatory signature. **A** Hierarchical clustering analysis revealed chromatin accessibility among Matrigel organoids, collagen sphere, and dFEnS. **B** Overlap of COL-UP (1880 genes) and FEnS-UP (1769 genes) with genes showing adjacent COL-OPEN (1169 sites) and FEnS-OPEN (1367 sites) is visualized respectively. The *p* value of statistical significance is indicated. **C** ATAC sequencing tracks illustrate dynamic chromatin opening at *Tead4*, *Wwtr1*, and *Tacstd2* in COL-ENRWN and dFEnS. The open chromatin region is highlighted with a red bar. **D** Top three enriched TF motifs in open chromatin of the collagen sphere and dFEnS identified by ATAC sequencing analysis are shown

In order to dissect an epigenetic aspect underlying cell fate conversion, we examined an open chromatin status in the collagen sphere, using the assay for transposase-accessible chromatin using sequencing (ATAC-seq) [16]. Firstly, according to the peak heat map, normalized ATAC-seq signal intensity showed no obvious difference among MG-ENR, COL-ENRWN, and dFEnS (Supplementary Fig. 2). However, hierarchical clustering indicated that COL-ENRWN and dFEnS were classified into the same cluster consistently with RNA-seq (Fig. 3A). To further analyze the potential relationship between chromatin accessibility alterations and DEGs, we assigned the different accessible regions (DARs) to the nearest mRNAs according to their genomic locations. As a result, DARs were strongly associated with upregulated genes in COL-ENRWN (*p*=2.0*10^-69^) and dFEnS (*p*=3.2*10^-58^) (Fig. 3B). In fact, ATAC-seq tracks illustrated that dynamic chromatin opening at *Tead4*, *Wwtr1*. Among fetal markers, *Tacstd2* [26] showed a significant chromatin opening (Fig. 3C), while other fetal marker genes such as *Ly6a* and *Clu* did not show distinct changes in chromatin status from MG-ENR (Supplementary Fig. 3). These results suggest the presence of complexity in addition to the chromatin accessibility alone in the induction of the reprogrammed signature.

To identify the candidate transcriptional factors (TFs) governing the remodeling program, we identified enriched motifs in sites of differential chromatin opening in COL-ENRWN and dFEnS in comparison with MG-ENR by ATAC-seq (Fig. 3D). The ATAC-based motif analysis revealed that top three TFs were the same in both COL-ENRWN and dFEnS. Not only the TEAD family, but also AP-1 transcriptional factor Fra1 and RUNX2, both of which are identified in enriched transcription in the collagen sphere, were enriched in COL-ENRWN and dFEnS, which suggests that the dynamic fate conversion of epithelial cells into a primitive state in COL-ENRWN might be orchestrated by the synergistic combination of AP-1, RUNX2, and TEAD.

### Application of collagen sphere to the human colon

**Fig. 4:**
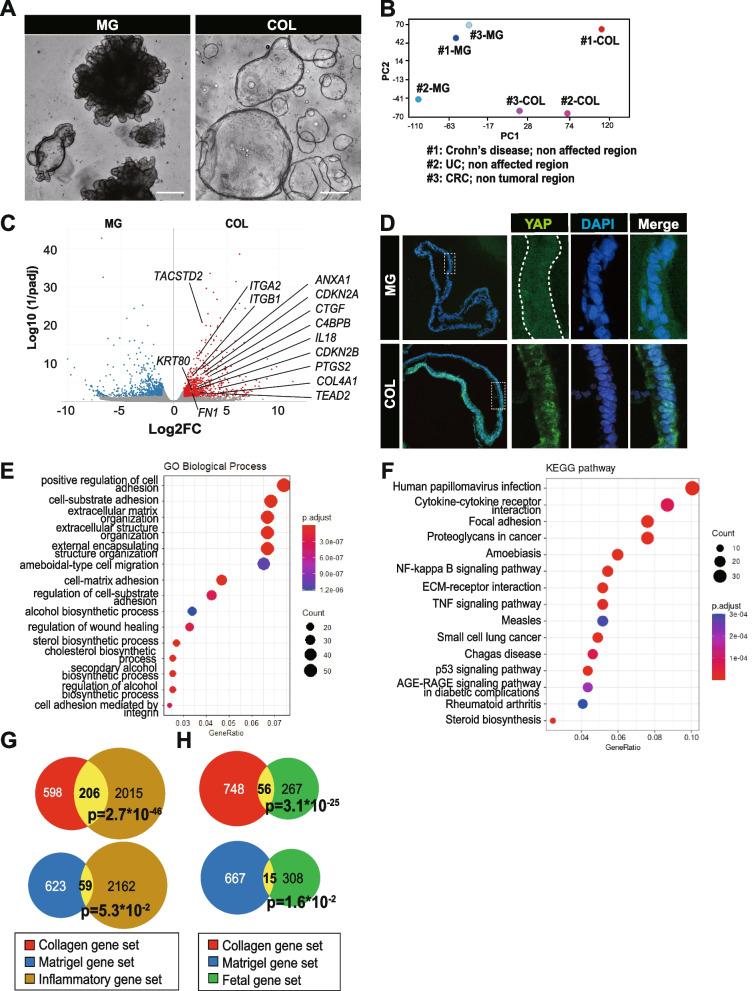
Application of collagen sphere to human colon. **A** Microscopic images of human colonic organoids cultured in Matrigel with WRN conditioned medium (left) and in COL with recombinant medium (right) are shown. Scale bars, 300 μm (left) and 200μm (right). **B** Principal component analysis of RNA-seq data from Matrigel organoids (MG) and collagen spheres (COL) derived from 3 different human colonic samples is shown. The plot represents 17,500 genes after discarding genes with low expression levels (<count16). Sample origins are indicated underneath the plot. **C** A volcano plot of DEGs (padj<0. 05, FC≥2) between MG and COL with DEGs indicated. Each plot indicates the average log 2-fold change (*x*-axis) and log 10 of 1/adjusted *p* value (*y*-axis) of triplicate samples of each condition. **D** Immunofluorescence images of YAP (green) of human colonic organoids in MG (top panel) and COL (bottom panel) are shown. Images are counterstained with DAPI (blue). A dashed square is enlarged in adjacent images for YAP, DAPI, and Merge. Scale bar, 50 μm. **E**, **F** Top 20 GO-TERM (BP direct) (**E**) and KEGG pathway (**F**) in upregulated gene set in COL compared to MG is shown. **G**, **H** Venn diagram depicting the overlap of COL gene set (804 genes; red and yellow) or MG gene set (682 genes; blue and yellow) with inflammatory gene set (*p*<0.05, FC≥2; adapted from ref [29]) (2221 genes; brown and yellow) (**G**) or fetal gene set (adapted from ref [30]) (323 genes; green and yellow) (**H**) is shown. The overlap is indicated with a number of genes (yellow). The *p* value of statistical significance is indicated

Subsequently, we evaluated whether the inflammatory signature could be also induced in the collagen sphere of the human colon epithelium. Isolated crypts were harvested from fresh surgical specimens of non-inflamed regions of Crohn’s disease and ulcerative colitis (UC) and of non-tumoral regions of colorectal cancer (CRC) (Table 2, #1-3). Firstly, harvested crypts were embedded in Matrigel and propagated by using EGF and L-WRN-conditioned medium as standard organoids, which have numerous parts of the protrusion (Fig. 4A, Table 1) [27]. After 2–3 passages, fragments of organoids were replated in collagen type I under the defined culture medium supplemented with several recombinant factors such as EGF, Noggin, R-spondin1, and Wnt3a as spherical structures, which is a resemblance to the collagen sphere of mouse intestine (Fig. 4A, Table 1). We identified DEGs by performing RNA sequencing analysis of Matrigel organoids and collagen spheres at passage 2 or 3 (Supplementary Table 4; Sheet 1). According to PCA in RNA-seq, the collagen sphere showed a distinct gene signature from Matrigel organoid (Fig. 4B). We identified 804 upregulated genes (FC≥2, padj<0.05; human COL gene set) and 682 downregulated genes (FC≤0.5, padj<0.05; human MG gene set) in the collagen sphere compared to Matrigel organoids among the 3 samples (Supplementary Table 4; sheet 2, 3). The human COL gene set includes genes such as *ANKRD1* (FC: 85), *COL4A1* (FC: 22), *PTGS2* (FC: 19), *C4BPB* (FC: 12), *CTGF* (FC: 8.4), *TACSTD2* (FC: 6.6), *ITGA2* (FC: 5.4), *TEAD2* (FC: 5.1), *ANXA1* (FC: 5.1), *CDKN2A* (FC: 4.9), *CDKN2B* (FC: 4.7), *IL18* (FC: 4.5), *KRT80* (FC: 3.7), *FN1* (FC: 3.4), and *ITGB1* (FC: 3.1) (Fig. 4C). Upregulation of *ITGA2* and *ITGB1* suggests the enhanced integrin-mediated adhesion to collagen type I has an impact on the specific property of human collagen sphere [28]. The expression of YAP, which acts as a key regulator in the process of cell fate conversion in murine organoid, was significantly elevated and nuclear translocation of YAP was retained in the human Collagen sphere (Fig. 4D). In GO TERM (biological process) analysis, positive regulation of cell adhesion, extracellular matrix organization, regulation of wound healing, cell adhesion medicated by integrin, etc., were related with human COL gene set (Fig. 4E). In KEGG enrichment analysis, focal adhesion, pathways in cancer, amoebiasis, NF-κΒ signaling pathway, ECM-receptor interaction, TNF signaling pathway, p53 signaling pathway, etc., were related with human COL gene set (Fig. 4F). These results illustrate that human colonic epithelial cells cultured in collagen type I is strongly associated with inflammation as in the case of mouse collagen sphere and that the reprogrammed signature can be induced also in human collagen sphere.

**Table 2:** Patient demographics: sample information is summarized in table

Patient ID	Disease	Region	Gross appearance	Gender
#1	Crohn disease	Transverse colon	Non-affected region	M
#2	Ulcerative colitis	Ascending colon	Non-affected region	M
#3	Colorectal cancer	Rectum	Non-tumoral region	M
#4	Ulcerative colitis	Descending/ascending colon	Colitis/non-inflamed	F

To verify if the human collagen sphere showed an inflammatory signature, we compared the gene sets to published transcriptomic datasets for the inflammatory epithelium of UC (inflammatory gene set, cutoff criteria: fold change≥2 and *p*<0.05) [29]. Two hundred six genes of the human COL gene set overlapped with the inflammatory gene set (*p*=2.7*10^-46^), whereas 59 genes of the human MG gene set did (*p*=5.3*10^-2^), suggesting that the human COL gene set showed a highly significant enrichment of inflammatory signature (Fig. 4G). Additionally, to verify if the human collagen sphere showed a fetal-like signature, we compared the gene sets to published mRNA datasets for human fetal colon epithelium (human fetal gene set) [30]. Fifty-six genes of the human COL gene set overlapped with the human fetal gene set (*p*=3.1*10^-25^), while 15 genes of the human MG gene set did (*p*=1.6*10^-2^), indicating that the human COL gene set showed higher similarity to the human fetal gene set (Fig. 4H). Based on these results, we think that collagen type I-dependent mechanotransduction can induce the inflammatory signature in epithelial cells in humans, capturing the fate conversion towards fetal progenitors.

### Hub genes in mouse and human collagen sphere identified in gene network analysis

**Fig. 5:**
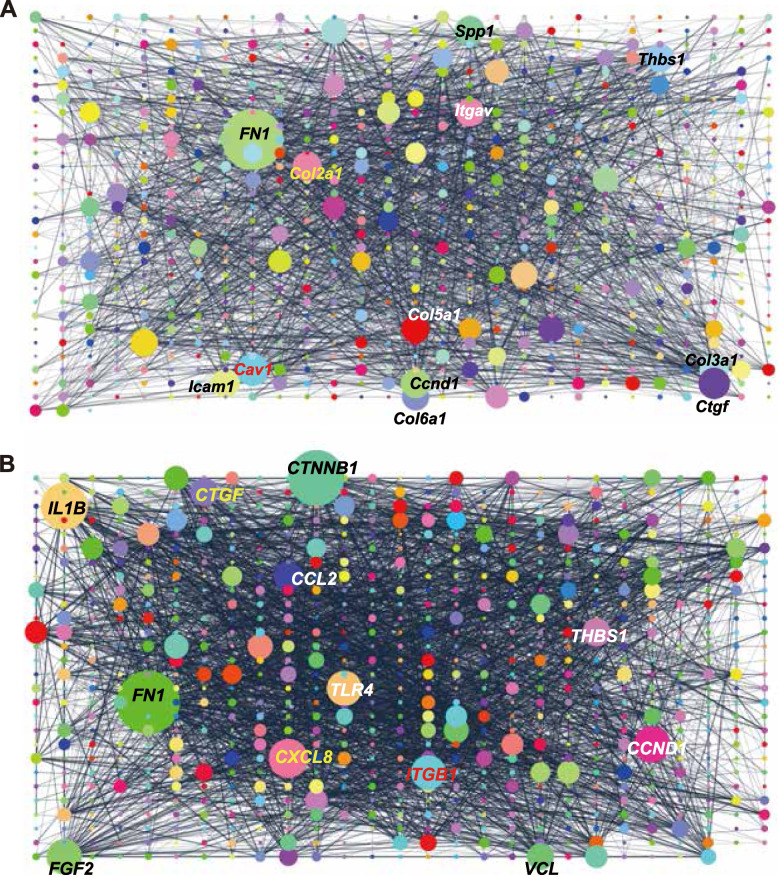
Hub genes in mouse and human COL sphere identified in gene network analysis,. **A**, **B** Mouse COL gene set (padj<0.05, FC≥4) compared to MG-ENR (**A**) and Human COL gene set (padj<0.05, FC≥2) compared to MG organoids (**B**) was analyzed in Cytoscape software using the String app. Each circle represents a hub node and the diameter of the circle corresponds with the degree of each node. Top 12 largest nodes are indicated with gene name

Importantly, the human COL gene set showed a significant overlap with the mouse COL gene set, which is newly narrowed down with new cutoff criteria of fold change≥4 and padj<0.05 (*p*=1.5*10^-51^) (Supplementary Fig. 4). To visualize the gene network in both gene sets, we performed gene network analysis in Cytospace using STRING app. Interestingly, *Fibronectin* was revealed to be the largest node in both mice (degree=89) and humans (degree=124). C*onnective tissue growth factor* (degree=43 in mice, 51 in humans), *Cyclin D1* (degree=40 in mice, 68 in humans), *thrombospondin1* (degree=39 in mice, 52 in humans) are also found in the top 12 largest nodes in both mice and human (Fig. 5A, B), verifying the presence of similar gene networks in mouse and human intestinal epithelial cells in response to a surrounding collagen type I niche. The second largest node in the human gene set is *CTNNB1* (degree=109), implying the synergistic effect with Wnt3a, which is the indispensable growth factor for successful collagen culture [10]. Other hub nodes ranked in the top 12 in mice are *Cav1* (degree=45), *Col1a2* (degree=40), *Col3a1* (degree=40), *Spp1* (degree=40), *Itgav* (degree=39), *Icam1* (degree=39), *Col5a1* (degree=38), and *Col6a1* (degree=37). In human, the rest of hub genes ranked in the top 12 are *IL1B* (degree=90), *CXCL8* (degree=74), *TLR4* (degree=66), *FGF2* (degree=66), *ITGB1* (degree=65), *CCL2* (degree=54), and *VCL* (degree=48).

### Histological validation of human COL gene set

**Fig. 6:**
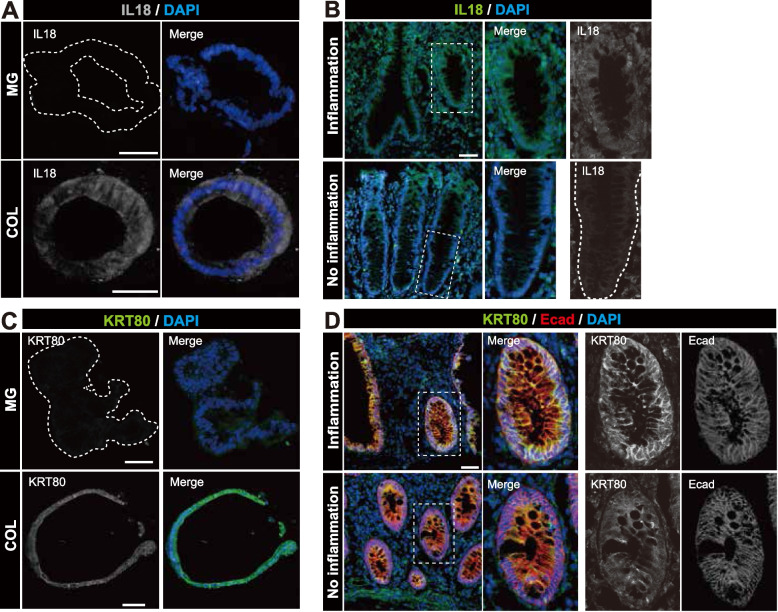
Histological validation of human COL gene set. **A** Immunofluorescence images of Il18 (gray) of human colonic organoids in Matrigel (MG, top) and in collagen (COL, bottom) are shown. In MG panel, the region of organoids is indicated with a dashed while line. In the right panels, merged images with DAPI (blue) are shown. Scale bars, 50 μm. **B** Immunofluorescence images of IL18 (green) in surgical specimen from inflamed (inflammation) and non-inflamed (no inflammation) regions of ulcerative colitis are shown. Images are counterstained with DAPI (blue). The insets magnify areas indicated by a dashed square with staining of IL18 (gray) alone. A white dashed line in an enlarged gray image of non-inflamed region indicates a crypt/lamina propria boundary. Scale bar, 50μm. **C** Immunofluorescence images of KRT80 (gray) of human colonic organoids in Matrigel (MG, top) and in collagen (COL, bottom) are shown. In MG panel, the region of organoids is indicated with a dashed white line. In the right panels, colored images (green) counterstained with DAPI (blue) are shown. Scale bars, 50 μm. **D** Immunofluorescence images of KRT80 (green) in surgical specimen from inflamed (inflammation) and non-inflamed (no inflammation) regions of ulcerative colitis are shown. Images are counterstained with E-cadherin (red) and DAPI (blue). The insets magnify areas indicated by a dashed square with the staining of KRT80 (gray) and E-cadherin (gray) alone. Scale bar, 50μm

It is important to see whether the observed fate conversion of intestinal epithelial cells is the recapitulation of inflammatory epithelium in vivo. To test this, we utilized samples obtained from the inflammatory and non-inflammatory regions of UC (Table 2, #4). Among identified genes, we validated the expression of IL18, as this is a general pro-inflammatory mediator known to be upregulated in IBD [31]. IF analysis revealed the upregulated protein expression in the cytoplasm in the collagen sphere compared to Matrigel organoids (Fig. 6A). Upregulation in the epithelial compartment of the active inflammatory region compared to no inflammatory region in UC pathological samples (Fig. 6B) is considered to be an in vivo phenotype. We also validate the expression of KRT80, which is included in both the human COL gene sets and inflammatory signature [29] and revealed that KRT80 is also upregulated in both the collagen sphere (Fig. 6C) and inflammation in vivo (Fig. 6D). Although the number of genes of human COL gene set is not fully elucidated in IBD research yet, a complement factor C4BPB, another gene found in the overlapped signature between human COL gene set and inflammatory signature, also showed the upregulated expression in inflammation (Supplementary Fig. 5), suggesting that collagen type I medicated fate conversion of epithelial cells dictates the induction of inflammatory signature also in human in vivo and that human COL gene set is a valid data set to understand the epithelial signature of inflammation in human IBD.

### Overview of collagen type I-mediated epithelial cell plasticity

**Fig. 7:**
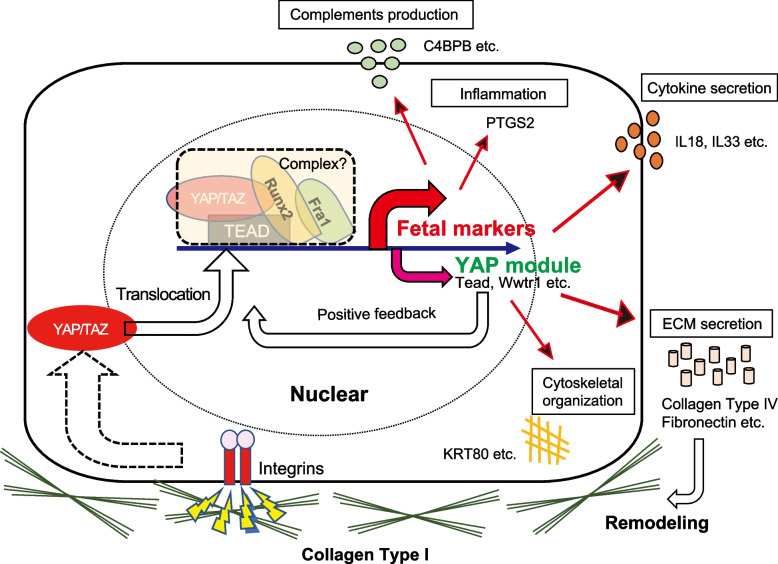
Overview of collagen type I-dependent epithelial cell plasticity. Schematic illustration how collagen type I triggers epithelial cell fate conversion in both mouse and human cells is presented. Collagen type I stimulates integrin a2b1 heterodimers, subsequently promotes nuclear translocation of YAP/TAZ. In the nuclear, YAP/TAZ coordinates with Fra1 and Runx2 to induce fetal marker genes such as *Ly6a/Clu/Tacstd2* in mouse and *TACSTD2* in human. Genes involved in YAP module such as *Tead/Wwtr1* are also induced, which will be subsequently recruited into the cascade to form a positive feedback loop. Downstream of the cascade, various responses are initiated, including the production of inflammatory molecules PTGS2, cytokine secretion such as IL33 and IL18, cytoskeletal organization with several different types of keratins such as KRT80, complements production such C4BPB, and ECM secretion such as collagen type IV and fibronectin. ECM secretion will subsequently remodel the ECM-niche and will influence the cascade

Finally, we summarize the overview of collagen type I-mediated epithelial fate plasticity (Fig. 7). In both mice and humans, collagen type I initiates YAP/TAZ activation via integrins, which subsequently induce a set of transcription including fetal markers such as *Ly6a*, *Clu*, and *Tacstd2* in mouse and *TACSTD2* in humans. This cascade would contain the feedback loops. Upregulation of *Wwtr1*, *Tead4*, *Fosl1*, and *Runx2* in mouse cells and *TEAD2* in human cells at the transcriptional level would enhance the cascade after being recruited to YAP/TAZ module. ECM molecules such as collagen type IV or fibronectin, which are elevated in both mice and humans at the transcriptional level, may remodel the ECM niche, which may also influence the cascade. At the same time, upregulation of tumor suppressor genes such as *Cdkn2a* in mice and *CDKN2A*/*CDKN2B* in humans may work as a negative feedback loop, which suppresses the exacerbating response to collagen type I stimuli. Cytokine secretion such as IL33 in mice [32] and IL18 in humans [31], and the production of PTGS2 [33] in both mice and humans matches the conventional epithelial properties of inflammation. Cytoskeletal remodeling by several keratins such as KRT80 in humans and the production of complements such as C4BPB in humans are also revealed to be a part of mechanotransduction pathway.

## Discussions

The activation of yes-associated protein 1 (YAP1) is reported to be important in the injury-dependent stem cell expansion process [6]. We previously revealed that the deposition of collagen matrix in mesenchyme triggers FAK/Src-mediated activation of YAP/TAZ in the epithelial compartment lying on the wound bed. That is, mechanotransduction pathways centered on the activation of YAP/TAZ act as a part of a regenerative cascade to induce the fetal-like reprogramming of intestinal epithelial cells [4]. Entire regulatory networks of the collagen sphere shown in this study support the concept that the occurrence of a fetal-like regenerating stem cell population coincides with ECM remodeling. In a word, the collagen sphere has properties of “inflammanoids” and retains YAP/TAZ-TEAD axis as a central module for induction of the distinctive signature. Considering that a growing body of evidence suggests that YAP/TAZ mechanotransduction is critical for driving tissue regeneration in various organs such as the liver, lung, heart, cornea, and skin [34–39], and elucidation of the upstream ECM-dependent regulatory mechanism to control YAP/TAZ is likely to have a widespread impact in various organs to understand the link between inflammation and regeneration.

In the current study, we confirmed that the serum-free culture system of the intestine/colon of mice and humans using purified collagen type I enabled it to induce the inflammatory profile and presented the detailed transcriptional and epigenetic landscape. Consistent with our previous study, the current study illustrated the upregulation of *ITGA2* and *ITGB1* in the human collagen sphere, supporting the scheme, in which integrin receptors are located upstream of the signal cascade. Considering that other types of gene-coding ECM molecules such as fibronectin and collagen type IV are highly upregulated, the physical property of the culture environment, such as stiffness or tension of surrounding ECM, is supposed to be quite unique in the collagen culture system, which may also tune YAP/TAZ level [22]. Particularly, as *fibronectin* is the largest hub node in both mouse and human COL spheres, it is a relevant role in regenerative cascade and must be an interesting future subject. Besides the critical role of YAP1, we identified that the cell fate conversion was associated with both transcriptional upregulation and dynamic changes in chromatin states of both *Wwtr1* and *Tead4*. Moreover, Fra1 and Runx2 were supposed to cooperate with YAP/TAZ-TEAD complex to promote the regenerative response of intestinal epithelium, though the formation of the transcriptional complex of these proteins must be validated in the future study. The study also highlighted that collagen type I-dependent reprogramming is a synergistic phenomenon with Wnt signal activation as being verified in gene network analysis in the human COL gene set, which extracts *CTNNB1* as the second largest hub node. These findings are critically important to understand the molecular mechanisms of how inflammation-associated remodeling of ECM in the mesenchyme affects the cellular properties of epithelial cells overlying the wound bed.

According to recent progress in the field, the regenerative process with transient activation of YAP1 can be recapitulated also in Matrigel organoids [40] and the existence of a regenerative fetal-like Ly6a+ stem cell population can be identified also in Matrigel organoids by single-cell RNA sequencing [7]; however, as we showed here, Matrigel organoids display the limited cellular plasticity. We think that the ECM component is one of the important cues to provide the relevant study model [41], even suitable for a drug screening under an adequate inflammatory environment. Considering that the analysis of distinctive fetal-like epithelial cell populations will become an increasingly important issue to dissect the repairing process of the intestinal epithelium and the property of inflammatory epithelium in the future, the usefulness of the collagen sphere no longer needs to be emphasized.

The establishment of the human collagen sphere with the activation of YAP/TAZ is also likely to have a widespread impact on pathology/pathophysiology commonly underlying in various types of human intestinal inflammation such as inflammatory bowel disease (IBD). This study identified a number of genes, which is not yet well elucidated in terms of their roles in inflammation and regeneration, however, we think that the gene set is a particularly informative data set to fully understand the inflammatory epithelial signature. For example, KRT80, one of the upregulated molecules in the collagen sphere, is known to be significantly upregulated in CRC tissues as compared with matched normal tissues [42]. Together with KEGG pathway analysis, which identified a pathway in cancer in activated pathways in the collagen sphere, it is postulated that there exists a link from regenerative cascade to colorectal carcinogenesis.

The entire landscape of epithelium in inflammation would be much more complicated due to the presence of the complicate interplays between mesenchymal and immune cells via several different types of cytokines [43, 44]; however, the results shown in this study clearly present the important role of collagen type I in triggering the epithelial fate change during inflammation towards regeneration.

## Conclusions

This study illustrated the precise molecular dynamics of collagen type I-mediated epithelial cell plasticity. Elucidation of the reprogramming mechanisms and its dependency on ECM-niche may bring a new insight to understand how inflammation triggers tissue regeneration via ECM remodeling and would provide a novel tool to control exacerbating inflammatory response.

## Supplementary Information

**Additional file 1: Supplementary Figure 1.** Collagen sphere has overlapped signature with Sca1 gene signature. **Supplementary Figure 2.** Quality of ATAC-seq analysis in the study. **Supplementary Figure 3.** ATAC-seq tracks of fetal markers. **Supplementary Figure 4.** Similarity of COL sphere between mouse and human. **Supplementary Figure 5.** C4BPB expression is upregulated in inflamed colonic epithelium.

**Additional file 2: Supplementary Table 1.** Microarray expression data in mouse organoids. Sheet 1: List of genes and its expression level in 4 samples (MG-ENR, MG-ENRWN, COL-ENRWN, dFEnS) after excluding the probe sets which failed to reach an expression value of 100 in at least one sample. Sheet 2: Genes upregulated in MG-ENRWN more than 2 folds change compared to MG-ENR. Sheet 3: Genes upregulated in COL-ENRWN more than 2 folds change compared to MG-ENR.

**Additional file 3: Supplementary Table 2.** RNA-seq expression data in mouse organoids. Sheet 1: List of genes and its expression level in 3 samples (MG-ENR, COL-ENRWN, dFEnS) in biological duplicate. Exclusion criteria is padj<0.1. Sheet 2: Genes upregulated in COL-ENRWN more than 2 folds change compared to MG-ENR. Sheet 3: Genes upregulated in dFEnS more than more than 2 folds change compared to MG-ENR.

**Additional file 4: Supplementary Table 3.** Results of ATAC-seq in mouse organoids. Sheet 1: List of DARs in 3 samples (MG-ENR, COL-ENRWN, dFEnS) in biological duplicate.

**Additional file 5: Supplementary Table 4.** RNA-seq expression data in human organoids. Sheet 1: List of genes and its expression ratio (COL/MG) after discarding genes of which RLE is 0 in all 6 samples. Sheet 2: Genes upregulated in COL sphere more than 2 folds change compared to MG organoids (cutoff criteria: padj<0.05).

## Data Availability

Expression array, RNA-seq, and ATAC-seq will be available from a corresponding author on reasonable request. Human organoids can be used only at TMDU according to the regulation of the study.

## Abbreviations

AP-1: Activator protein1
ATAC-seq: Assay for transposase-accessible chromatin using sequencing
COL: Collagen type I
CRC: Colorectal cancer
DARs: Different accessible regions
DEGs: Differentially expressed genes
dFEnS: Fetal intestinal organoids from distal part of small intestine
DSS: Dextran sulfate sodium
ECM: Extracellular matrix
E16: Embryonic day 16
FEnS: Fetal enterosphere
FC: Fold change
FPKM: Fragments per kilobase of transcript per million mapped reads
GO: Gene Ontology
IBD: Inflammatory bowel disease
KEGG: Kyoto Encyclopedia of Genes and Genomes
MG: Matrigel
padj: Adjusted *p* value
PBS: Phosphate buffer saline
PCA: Principal component analysis
Sca1: Stem cell antigen-1
TFs: Transcriptional factors
TPM: Transcripts per million
UC: Ulcerative colitis
YAP1: Yes-associated protein 1
