# Supplementary material for: Collagen type I-mediated mechanotransduction controls epithelial cell fate conversion during intestinal inflammation
Source: Inflamm Regen. 2022 Nov 28;42:49. doi: 10.1186/s41232-022-00237-3 (PMC9703763; doi:10.1186/s41232-022-00237-3)
Supplement: Supplementary file 1 — Additional file 1: Supplementary Figure 1. Collagen sphere has overlapped signature with Sca1 gene signature. Supplementary Figure 2. Quality of ATAC-seq analysis in the study. Supplementary Figure 3. ATAC-seq tracks of fetal markers. Supplementary Figure 4. Similarity of COL sphere between mouse and human. Supplementary Figure 5. C4BPB expression is upregulated in inflamed colonic epithelium. [file 41232_2022_237_MOESM1_ESM.pdf]

A

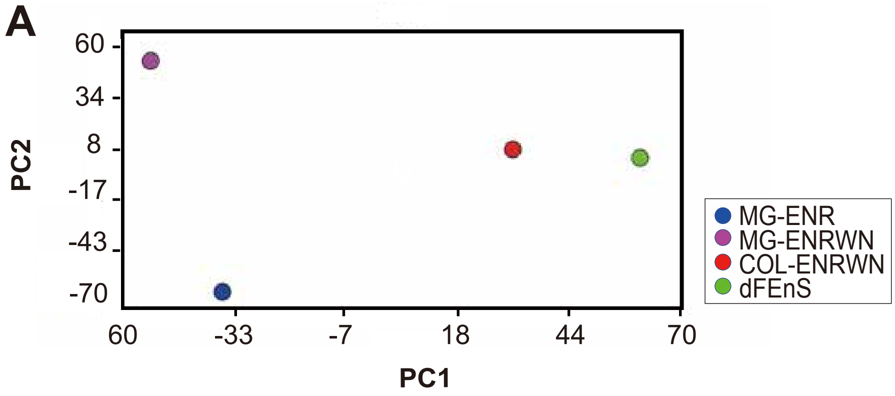

B

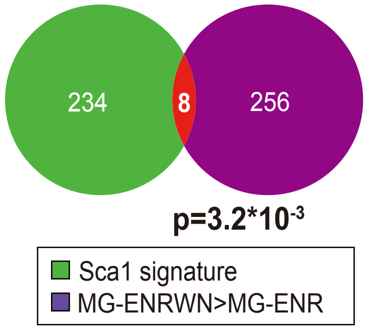

C

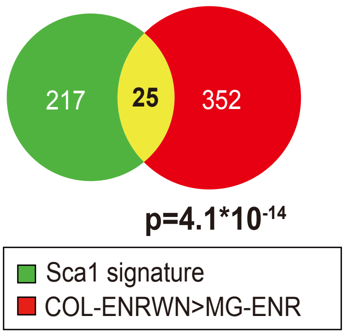

B’

Gene symbol
B4galnt1
Ly6a
Ly6c2
Ly6d
Mmp7
Nfkbia
Reg3b
Reg3g

C’

Gene symbol
Cd44
Clu
Ctgf
Cxcl16
D17H6S56E-5
Ddah1
Efemp1
Epha2
F3
Hsph1
Ier3
Ly6a
Ly6c2
Ly6d
Myl7
Nfkbia
Plat
Plaur
Plk2
Rbp1
Rdh10
Ripk3
Scd1
Sulf2
Tnfrsf12a

Supplementary Figure 1. Collagen sphere has overlapped signature with Sca1 gene signature.
(A) Principal component analysis of MG-ENR, MG-ENRWN, COL-ENRWN and dFEnS based on 6,597 variably expressed genes, of which expression value is >100, is shown.
(B) Venn diagram depicts the overlap between Sca1 signature and genes enriched in MG-ENRWN compared to MG-ENR (MG-ENRWN>MG-ENR) more than 2 folds. (B’) 8 genes which are commonly upregulated between Sca1 signature and MG-ENRWN>MG-ENR are listed.
(C) Venn diagram depicts the overlap between Sca1 signature and genes enriched in COL-ENRWN compared to MG-ENR (COL-ENRWN>MG-ENR) more than 2 folds. (C’) 25 genes which are commonly upregulated between Sca1 signature and COL-ENRWN>MG-ENR are listed.

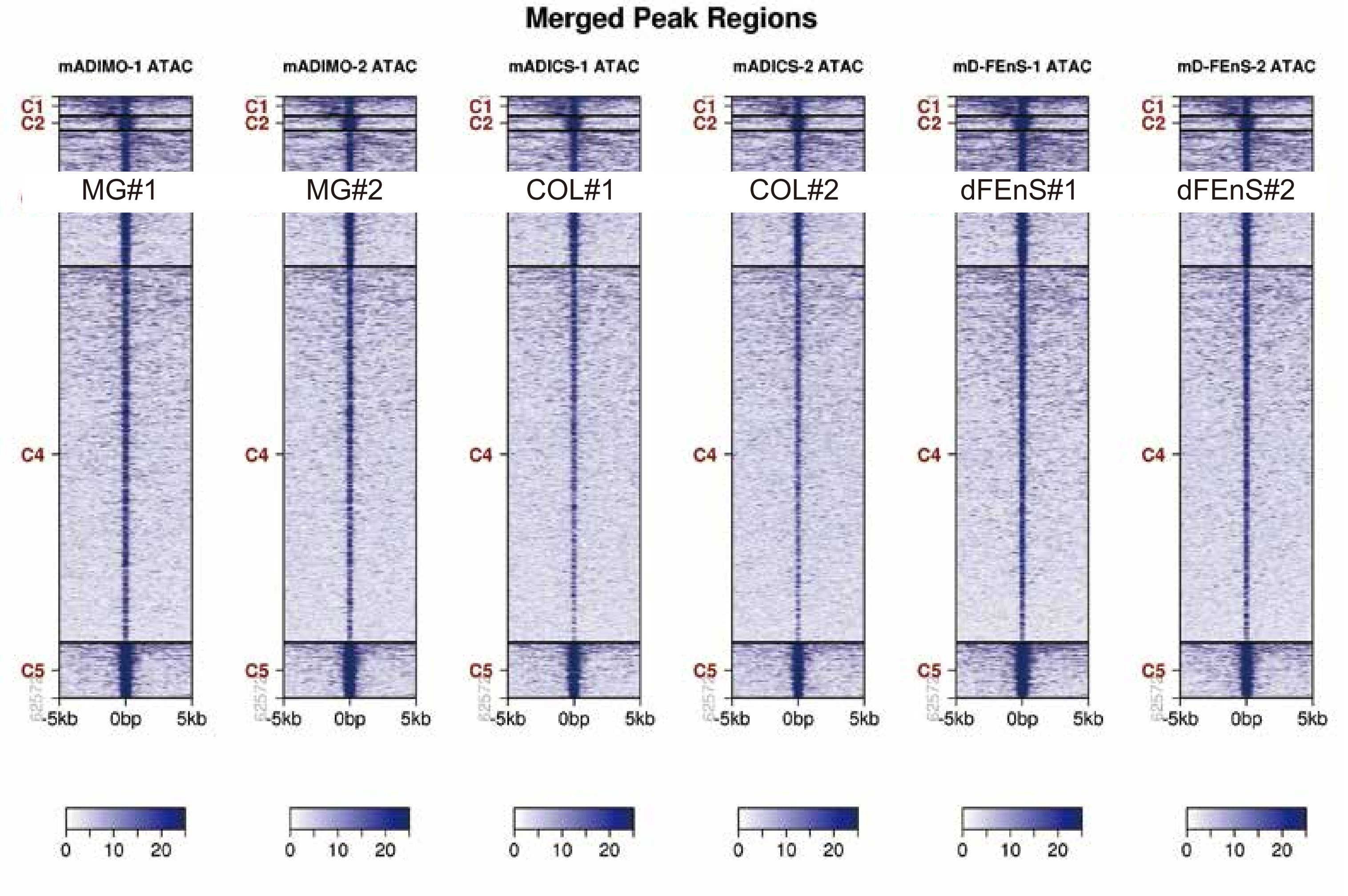

Supplementary Figure2. Quality of ATAC-seq analysis in the study

Peaks heat map shows normalized ATAC-seq signal among MG organoids, collagen sphere and dFEnS.

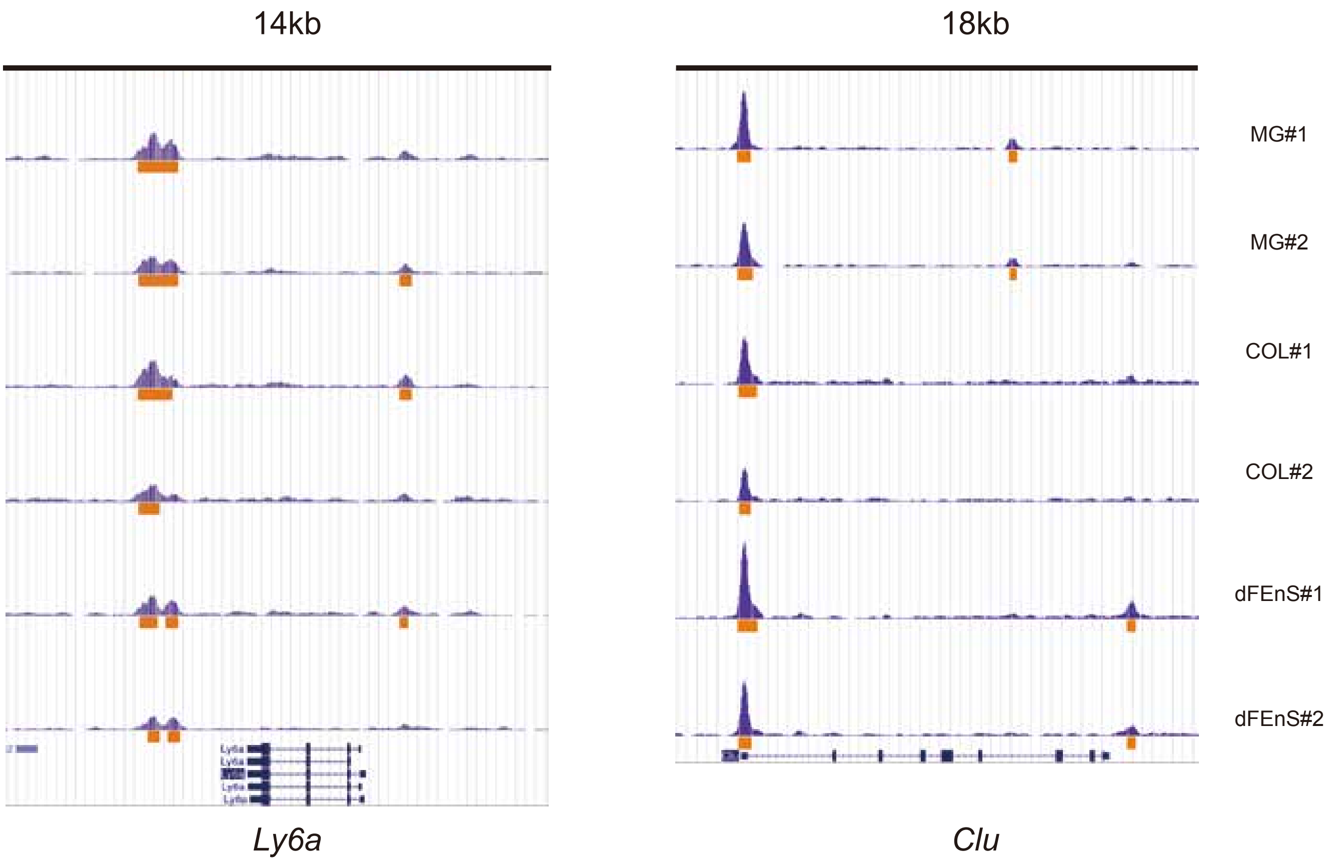

Supplementary Figure 3. ATAC-seq tracks of fetal markers

ATAC-seq tracks illustrate dynamic chromatin opening at *Ly6a* and *Clu*.

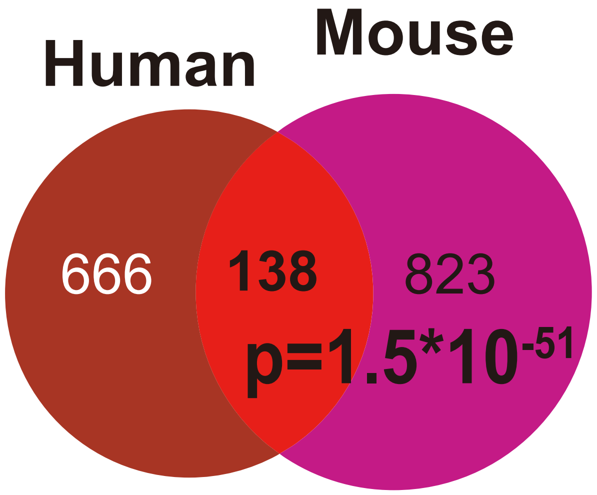

Supplementary Figure 4. Similarity of COL sphere between mouse and human

(A) Upregulated genes in human COL sphere comared to MG organoids (804 genes; fold change≥2, padj<0.05) showed a significant high overlap with upregulated genes in mouse COL sphere compared to MG-ENR (961 genes; fold change≥4,padj<0.05). The p-value of statistical significance is indicated.

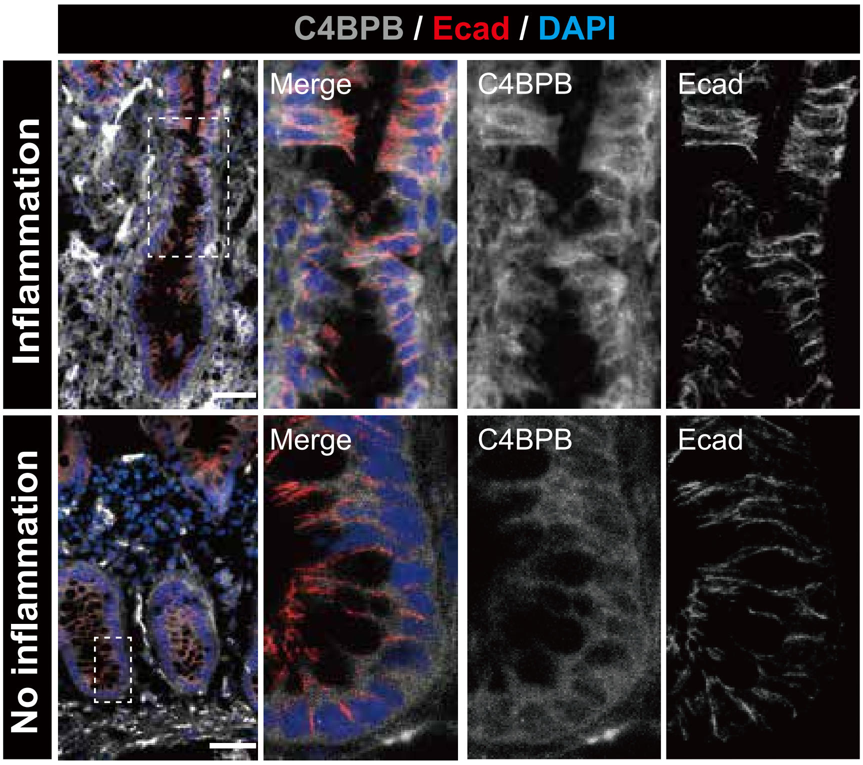

Supplementary Figure 5. C4BPB expression is upregulated in inflamed colonic epithelium.

Representative confocal immunofluorescence images of surgical specimen from inflamed region (inflammation) and non-inflamed region (No inflammation) obtained from a patient with ulcerative colitis stained with C4BPB (gray), E-cadherin (red) and DAPI (blue) are shown. Scale bar, 50μm. The insets magnify areas indicated by a dashed square with the staining of C4BPB (gray) and E-cadherin (gray), respectively.
